# Newborn Screening for Severe Primary Immunodeficiency Diseases in Sweden—a 2-Year Pilot TREC and KREC Screening Study

**DOI:** 10.1007/s10875-016-0347-5

**Published:** 2016-11-21

**Authors:** Michela Barbaro, Annika Ohlsson, Stephan Borte, Susanne Jonsson, Rolf H. Zetterström, Jovanka King, Jacek Winiarski, Ulrika von Döbeln, Lennart Hammarström

**Affiliations:** 1 Centre for Inherited Metabolic Diseases, Karolinska University Hospital Solna, SE-17176 Stockholm, Sweden; 2 Department of Molecular Medicine and Surgery, Karolinska Institutet, SE-17176 Stockholm, Sweden; 3 Department of Medical Biochemistry and Biophysics, Division of Molecular Metabolism, Karolinska Institutet, SE-17177 Stockholm, Sweden; 4 Department of Clinical Immunology, Karolinska University Hospital Huddinge, SE-14186 Stockholm, Sweden; 5 ImmunoDeficiencyCenter Leipzig (IDCL) at Hospital St. Georg Leipzig, Delitzscher Strasse 141, 04129 Leipzig, Germany; 6 Department of Immunopathology, SA Pathology, Women’s and Children’s Hospital Campus, North Adelaide, South Australia 5006 Australia; 7 Robinson Research Institute and Discipline of Paediatrics, School of Medicine, University of Adelaide, North Adelaide, South Australia 5006 Australia; 8 Department of Clinical Technology and Intervention, Karolinska Institutet, SE-14186 Stockholm, Sweden; 9 Department of Pediatrics, Karolinska University Hospital Huddinge, SE-14186 Stockholm, Sweden

**Keywords:** Newborn screening, primary immunodeficiency diseases, severe combined immunodeficiency, TREC, KREC

## Abstract

Newborn screening for severe primary immunodeficiencies (PID), characterized by T and/or B cell lymphopenia, was carried out in a pilot program in the Stockholm County, Sweden, over a 2-year period, encompassing 58,834 children. T cell receptor excision circles (TREC) and kappa-deleting recombination excision circles (KREC) were measured simultaneously using a quantitative PCR-based method on DNA extracted from dried blood spots (DBS), with beta-actin serving as a quality control for DNA quantity. Diagnostic cutoff levels enabling identification of newborns with milder and reversible T and/or B cell lymphopenia were also evaluated. Sixty-four children were recalled for follow-up due to low TREC and/or KREC levels, and three patients with immunodeficiency (Artemis-SCID, ATM, and an as yet unclassified T cell lymphopenia/hypogammaglobulinemia) were identified. Of the positive samples, 24 were associated with prematurity. Thirteen children born to mothers treated with immunosuppressive agents during pregnancy (azathioprine (*n* = 9), mercaptopurine (*n* = 1), azathioprine and tacrolimus (*n* = 3)) showed low KREC levels at birth, which spontaneously normalized. Twenty-nine newborns had no apparent cause identified for their abnormal results, but normalized with time. Children with trisomy 21 (*n* = 43) showed a lower median number of both TREC (104 vs. 174 copies/μL blood) and KREC (45 vs. 100 copies/3.2 mm blood spot), but only one, born prematurely, fell below the cutoff level. Two children diagnosed with DiGeorge syndrome were found to have low TREC levels, but these were still above the cutoff level. This is the first large-scale screening study with a simultaneous detection of both TREC and KREC, allowing identification of newborns with both T and B cell defects.

**Electronic supplementary material:**

The online version of this article (doi:10.1007/s10875-016-0347-5) contains supplementary material, which is available to authorized users.

## Introduction

The purpose of neonatal screening is the early recognition of treatable, mostly genetically determined diseases that manifest with a high rate of morbidity and mortality. Mass screening of newborn infants with dried blood spots (DBS) started in the early 1960s based on a method developed by Guthrie and Susi for diagnosing phenylketonuria [1]. Peripheral blood from a heel stick was blotted onto filter paper (referred to as “Guthrie card”) at 3 to 5 days after birth, dried, and sent by mail to centralized laboratories for analysis.

The US congress decided in 2006 to include 29 core and 25 secondary disorders in its newborn screening program based on a priority list of disorders. In recent years, a number of additional disorders, among them severe combined immunodeficiency (SCID), have been included in this list, based on the well-recognized Wilson and Jungner criteria [2]. In Sweden, neonatal screening with DBS was initiated in 1965 and today, a total of 24 disorders are included in the program.

Genetically determined disorders of immunity are commonly referred to as primary immunodeficiencies (PID) and were first recognized clinically over 60 years ago with the identification of X-linked agammaglobulinemia (XLA) [3]. Today, the group of PID includes more than 250 distinct entities, which are divided into T cell deficiencies, B cell deficiencies (the predominant group), combined T and B cell deficiencies, complement defects, and granulocyte defects [4].

Primary immunodeficiencies as a group are not rare diseases, but should be considered in all patients presenting with severe, atypical, or recurrent infections. The overall prevalence is unknown, but varies greatly (from 1:600 for IgA deficiency, 1:20,000 for common variable immunodeficiency, 1:50,000 for SCID, and 1:100,000 for XLA) [4] and references therein [5]. The estimated incidence of severe forms of PID, which require immediate attention, is variable in different populations, but would be expected to be in the order of 5–10 per 100,000 live births.

In 2005, Chan and Puck published a seminal paper, describing the T-cell receptor excision circle (TREC) assay as a tool for large-scale screening for SCID and other T cell lymphopenias [6]. T cell receptor genes are normally edited during T cell differentiation, and the deleted fragments are circularized and do not undergo further replication in dividing cells. Thus, TRECs are a marker of recently formed T cells. The TREC copy number can be determined using a quantitative PCR-based method using DNA extracted from routinely collected DBS. The TREC assay was implemented in the state of Wisconsin in 2008 [7] and its inclusion in the newborn screening programs in the USA was recommended in 2010. Currently, 38 states are screening for SCID using the TREC assay, with the remaining 12 states due to commence in 2017 (Jeffrey Modell Foundation, http://www.info4pi.org/). More than three million newborns have been tested to date [8]. The results show a frequency of SCID of 1/58,000 children (overall 1/7300 with clinically relevant forms of T cell lymphopenia) and a high survival rate (92 %) after treatment.

Congenital B cell lymphopenia can be identified by screening for kappa-deleting recombination excision circles (KREC), the circular by-product of B cell immunoglobulin kappa gene rearrangement [9]. Patients with severe forms of B cell deficiency such as XLA are easily identified by the KREC assay [10] and are treated with regular gammaglobulin infusions, thereby allowing a near to normal life. Furthermore, it has been shown that delayed-onset forms of SCID and other severe forms of PID that present with normal T cell numbers and TREC levels above cutoff in newborn screening programs may be identified using a combined TREC and KREC screening approach [11].

We thus initiated a screening program for all newborns in the county of Stockholm in Sweden using a combined TREC and KREC assay [12]. We previously analyzed 10,058 samples as part of a feasibility study, and one XLA patient was identified (Bruton tyrosine kinase gene mutation, c.1480C>T, p.Gln494Ter), thus demonstrating proof of principle of the combined screening approach [12]. Although a small cohort of Spanish newborns have been screened using the combined TREC/KREC assay [13], this represents the first large-scale prospective study where both T and B cell defects (lymphopenia) can be identified and the results of the first 2 years of the screening program are reported here.

## Materials and Methods

### Sampling

We carried out a study encompassing samples from all children born in the Stockholm County from November 15, 2013 to November 15, 2015, searching for children with T and B cell lymphopenia, in order to identify those with severe forms of PID. A total of 58,834 samples were analyzed. For 24 additional infants, the parents chose not to participate in the study. The blood sample was collected onto Whatman 903 filter paper as soon as possible after 48 h of age, presently at a mean age of 2.8 days, and was then mailed to the laboratory. The average age at recall for a positive screening result was 6 days.

The regional ethical board in Stockholm approved the study (Ethical permit 2013/414-31/4).

### TREC/KREC Screening Assay

The TREC/KREC newborn screening assay described previously [12] was utilized, with minor technical modifications. Briefly, DNA was extracted from single 3.2-mm punches of the original DBS in a 96-well format, and quantitative triplex real-time qPCR for TREC, KREC, and beta-actin (ACTB) was performed using a ViiA7 Real-Time PCR System (Applied Biosystems, Foster City, CA, USA) on DNA eluates as previously described [12]. The qPCR procedure was optimized using custom reagents provided by Affymetrix (Santa Clara, CA, USA). TREC and KREC levels were determined per 3.2-mm punch. Amplification of ACTB was used to assess that a sufficient quantity of DNA was extracted from each DBS, but this was disregarded if TREC and KREC copy numbers were above the cutoff level. The samples were initially analyzed once, and if the value was below the cutoff, they were reanalyzed in duplicate. Samples in which TREC and/or KREC levels were below cutoff in association with a reduction in ACTB copy number were considered inconclusive. Cutoff levels for TREC/KREC were adjusted during the study based on the yield of positive samples.

### Genetic Characterization of Patients with Low TREC/KREC Levels

For the three children with suspected PID, whole exome sequencing (WES) was performed, followed by confirmatory Sanger sequencing. DNA purification, library preparation, read mapping, variant determination, and analysis of WES data were performed as described previously [14, 15]. Sequences were generated as 90-bp pair-end reads and aligned to the human genome reference (UCSC hg 19 version, build 37.1) by using the SOAP aligner (soap 2.21) software. Duplicated reads were filtered out and only uniquely mapped reads were kept for subsequent analysis. The SOAPsnp (version 1.03) software was subsequently used with default parameters to assemble the consensus sequence and call genotypes in target regions. For single nucleotide polymorphism (SNP) quality control, low-quality SNPs that met one of the four following criteria were filtered out: (1) a genotype quality of less than 20, (2) a sequencing depth of less than 4, (3) an estimated copy number of more than 2, and (4) a distance from the adjacent SNPs of less than 5 bp. Small insertions/deletions were detected by using the Unified Genotype tool from GATK (version v1.0.4705) after alignment of quality reads to the human reference genome using BWA (version 0.5.9-r16).

### Statistical Analyses

Statistical analyses were performed using GraphPad Prism Version 6.0 (GraphPad Software, San Diego, CA). Given the nonparametric distribution of the data, the Mann-Whitney *U* test was used for group comparison analyses, and the Spearman correlation coefficient was calculated for correlation analyses. Differences were considered statistically significant when the *p* value was less than 0.05.

## Results

### Screening Overview

**Fig. 1:**
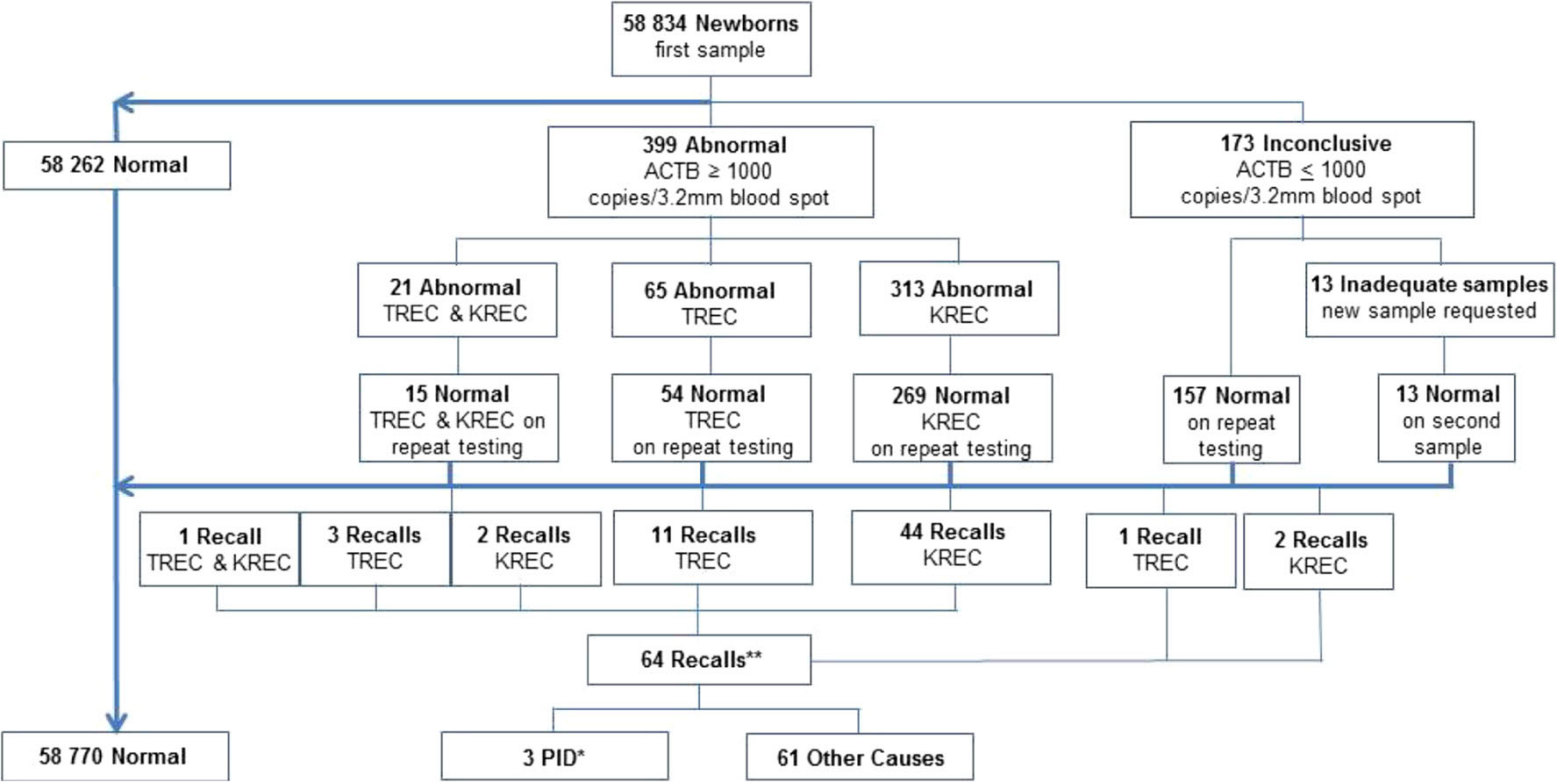
Summary of all newborn screening results between 15 November 2013 and 15 November 2015. *Two infants had low TREC levels, and one had low TREC and KREC levels. **See Fig. 2

In total, 58,834 newborns were screened. Samples with TREC and/or KREC copies below the cutoff values were considered “abnormal” (positive), whereas those that also had reduced ACTB copy numbers were considered to be “inconclusive.” Initially, 572 samples were found to be either abnormal (positive) or inconclusive (*n* = 399 and *n* = 173, respectively) (Fig. 1). After repeat testing on the original DBS, 64 abnormal (positive) samples remained, i.e., 99.9 % were considered normal and were not evaluated further. A total of 13 patients were recalled for repeat sampling due to poor quality of their initial DBS sample.

The definition of “low” levels of TREC and KREC copies will determine how many samples are considered positive. The dilemma is to avoid unnecessary recalls while still capturing all patients with a severe form of PID (i.e., SCID and XLA), as well as additional patients with profound disturbance of T and B cell homeostasis at birth. Three different cutoff levels for recall were used during the study as the limits were adjusted based on the number and outcome of the recalls. During the first period, a cutoff level of 15 copies/3.2 mm blood spot or below for TREC and 10 copies/3.2 mm blood spot blood or below for KREC was applied. In total, 16,582 children were screened and 35 were considered to have a positive result. However, the vast majority recovered spontaneously (Supplementary Table 1) and the cutoff was subsequently lowered to a TREC level of 8 copies/3.2 mm blood spot or below and a KREC level of 4 copies/3.2 mm blood spot blood or below. Another 28,298 children were screened during this period and 11 were found to be positive, among them PID patients 1 and 2. As these cutoffs were ultimately considered too stringent, they were finally changed to a TREC level of 10 copies/3.2 mm blood spot or below and a KREC level of 6 copies/3.2 mm blood spot or below. Altogether, 13,954 children were subsequently screened and 18 were found to be positive, among them PID patient 3.

The patients with TREC/KREC levels below the cutoff (Supplementary Table 1) were referred to a pediatrician specialized in the diagnosis and management of PID. The patient with absent TREC and KREC was immediately hospitalized whereas in the vast majority of patients, a follow-up sample for TREC and KREC analysis was collected within 2–10 weeks (Table 1) depending on the clinical urgency and gestational age. The recall was almost exclusively due to low KREC levels, and these patients were usually seen and resampled at around 3 weeks of age. In the majority of cases, the TREC/KREC values had normalized at the time of resampling (Supplementary Table 1) and only three patients (approximately 1/20,000) were referred for flow cytometric (fluorescence-activated cell sorting, FACS) analysis and genetic work-up (WES). In the majority of recalled patients, follow-up investigation with FACS analysis was not performed, as it was not deemed clinically relevant. To date, none of the infants in whom KREC/TREC levels normalized have subsequently developed features of PID.

**Table 1:** Serial TREC and KREC levels in PID patients with abnormal screening test results

				TREC copies/3.2 mm blood spot				KREC copies/3.2 mm blood spot			
Case	Sex	Gestational age (weeks)	Diagnosis	Result 1	Result 2	Result 3	Result 4	Result 1	Result 2	Result 3	Result 4
1	Male	34	Artemis deficiency	Day 2 0	Day 8 0	Day 14 0	Day 24 0	Day 2 0	Day 8 0	Day 14 0	Day 24 0
2	Male	39	Ataxia-telangiectasia	Day 2 5	Day 25 5	Day 43 3	Day 51 3	Day 2 7	Day 25 10	Day 43 21	Day 51 19
3	Male	36	Unknown genetic defect^a^	Day 2 7	Day 27 4	Day 34 5	Day 94 1	Day 2 205	Day 27 732	Day 34 504	Day 94 250

^a^T cell lymphopenia and hypogammaglobulinemia. At follow-up at 15 months of age, the child’s hypogammaglobulinemia had resolved but a persistent idiopathic CD3+ T cell lymphopenia (absolute CD3+ count = 0.9 × 10^9^/L) was evident

### Abnormal (Positive) TREC/KREC Results

**Fig. 2:**
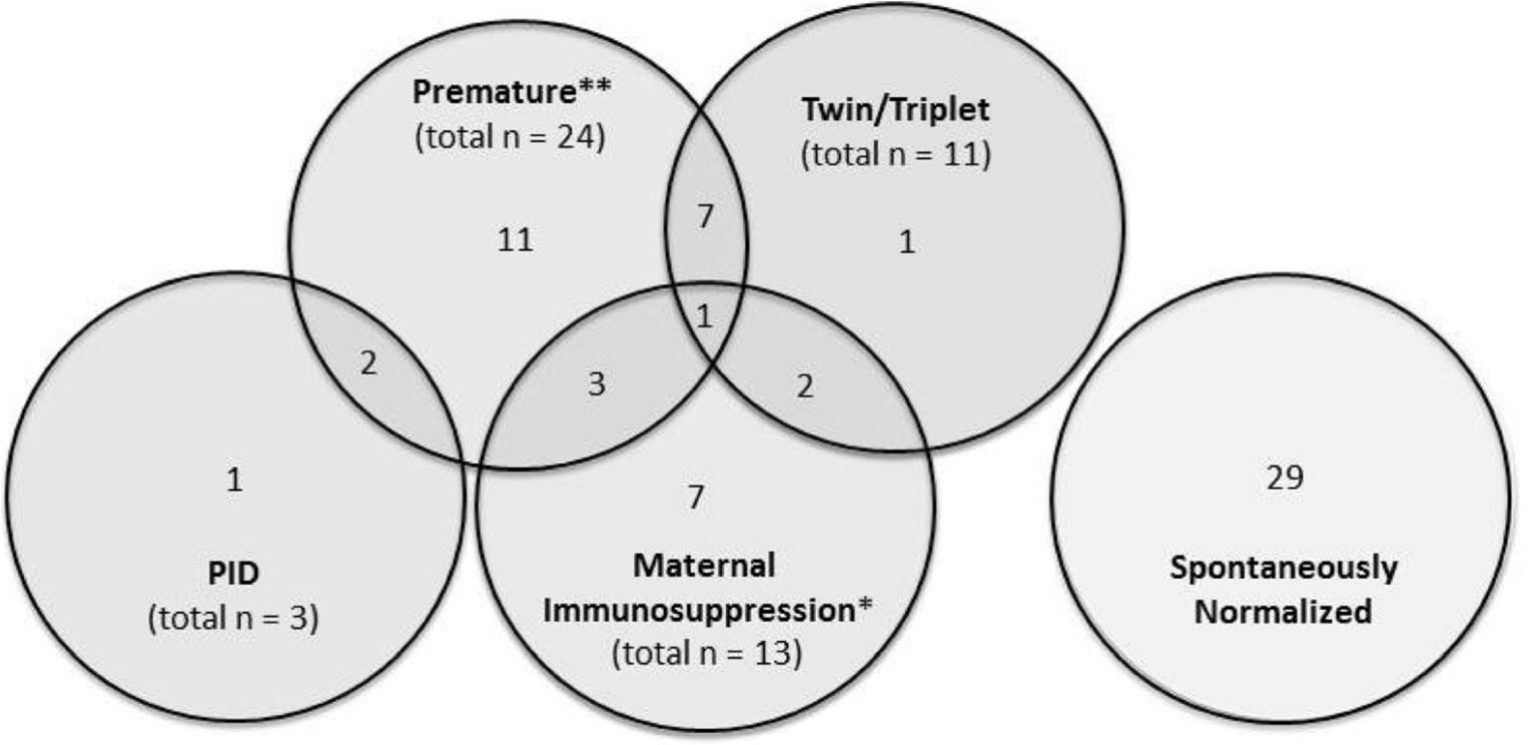
Characteristics of the 64 infants with abnormal screening results recalled for repeat testing. *Azathioprine (*n* = 9), mercaptopurine (*n* = 1), azathioprine + tacrolimus (*n* = 3). **One infant who was premature also had trisomy 21

Altogether, 64 children were recalled for follow-up due to low TREC and/or KREC levels and three patients with severe primary immunodeficiency were identified (Supplementary Table 1). Among the positive samples (Fig. 2), several infants had more than one potential etiological factor contributing to their low TREC and/or KREC levels. A total of 24 infants were premature, including one with trisomy 21 and 11 were a twin or triplet. Furthermore, 13 infants were born to mothers receiving immunosuppressive therapy. Twenty-nine had no apparent cause identified and the TREC/KREC levels in the children tested (*n* = 27) normalized with time. Resampling was declined for four of the 64 children recalled.

The 13 children born to mothers who were treated with immunosuppressive agents during pregnancy (azathioprine (*n* = 9), mercaptopurine (*n* = 1), azathioprine and tacrolimus (*n* = 3)) showed low KREC levels at birth, which however spontaneously normalized after 2–10 weeks. Two children from a mother treated with azathioprine, born almost 2 years apart, both showed low KREC at birth.

### Other Conditions Influencing the Screening Result

Children with trisomy 21 (*n* = 43) showed a lower median number of both TREC (104 vs. 174 in the total material) and KREC (45 vs. 100), but only one child, born prematurely, fell below the cutoff levels applied at the time of testing. Two children were diagnosed with DiGeorge syndrome in the Department of Clinical Genetics, and both had a low TREC level but these were still above the cutoff level at the time of testing.

**Fig. 3:**
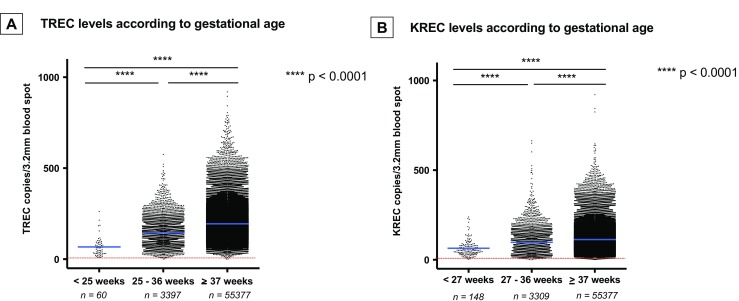
Comparison of TREC levels (**a**) in infants born prior to 25 weeks, between 25 and 36 weeks, and at term (≥37 weeks) gestation. Comparison of KREC levels (**b**) in infants born prior to 27 weeks, between 27 and 36 weeks, and at term (≥37 weeks) gestation. Each *point* represents one infant and the number of infants in each group is indicated. The *horizontal blue line* indicates the mean value of all samples. The *dashed horizontal red line* represents the cutoff value for TREC (<10 copies/3.2 mm blood spot) and KREC (<6 copies/3.2 mm blood spot) levels, respectively. ****p < 0.0001, Mann-Whitney *U* test

Among the 3457 infants born prematurely (prior to 37 weeks gestation), 24 screened positive with low TREC (*n* = 12), KREC (*n* =11) levels, or both (*n* = 1) (Supplementary Table 1). Although T cells appear to mature late in fetal life, TREC and KREC levels were surprisingly “normal” in the majority of children, even at very low gestational ages (Table 2), albeit as a group they were still significantly below the levels in children born at term (*p* < 0.0001, Mann-Whitney *U* test) (Fig. 3).

**Table 2:** Mean TREC and KREC levels at different gestational ages

Gestational age (weeks)	Infants (*n*)	Mean TREC level (copies/3.2 mm blood spot)	Mean KREC level (copies/3.2 mm blood spot)
20	1	262	139
21	2	97	180
22	3	84	97
23	23	53	52
24	31	68	58
25	38	64	70
26	50	71	62
27	58	89	65
28	53	93	76
29	97	114	91
30	97	139	110
31	122	134	99
32	199	146	90
33	283	139	87
34	390	142	93
35	663	153	97
36	1347	156	103
Total	3457

**Fig. 4:**
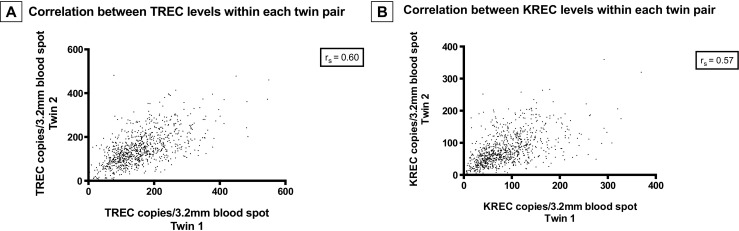
Correlation between TREC (**a**) and KREC (**b**) levels within all 896 twin pairs. Each *point* represents one twin pair, where the TREC value for twin 1 (*x axis*) is plotted against the TREC value for twin 2 (*y axis*) (**a**). KREC levels for each pair are similarly depicted (**b**). Overall, there was poor correlation between specimen pairs, with a calculated Spearman correlation coefficient (*R*_s_) of 0.6 for TREC and 0.57 for KREC (*p* < 0.0001)

A total of 896 twin pairs and 16 triplet sets (*n* =1840) were identified in the cohort, and the TREC and/or KREC values differed markedly between the newborns in both those siblings with low levels (Table 3) and in those with values within the normal range (Fig. 4).

**Table 3:** Discrepant TREC and KREC levels in twin and triplet sets with abnormal screening results

	Gestational age (weeks)	Sex	Twin/triplet	Age at first sampling (days)	TREC copies/3.2 mm blood spot	KREC copies/3.2 mm blood spot	Comments
1a^a^	38	Female	TW I	3	1	18	Deceased
1b	38	Female	TW II	3	138	161	
2a	34	Male	TW I	2	51	259	
2b^a^	34	Male	TW II	2	13	92	
3a^a^	25	Female	TW I	0	5	25	Deceased
3b	25	Female	TW II	0	19	178	
4a	29	Male	TR I	3	20	42	
4b^a^	29	Female	TR II	3	15	29	
4c	29	Female	TR III	3	70	56	
5a	29	Male	TW I	2	76	130	
5b^a^	29	Male	TW II	2	5	16	
6a	27	n.a	TR I				Deceased
6b	27	n.a	TR II				Deceased
6c^a^	27	Male	TR III	3	5	28	
7a^a^	38	Female	TW I	3	149	6	
7b^a^	38	Female	TW II	3	132	5	
8a	32	Female	TW I	2	77	40	
8b^a^	32	Female	TW II	2	13	5	
9a	24	Male	TW I	2	16	68	
9b^a^	24	Female	TW II	2	9	13	
10a	23	n.a	TW I				Deceased
10b^a^	23	Female	TW II	2	7	11	

*TW* twin 1, *TR* triplet, *n.a*. not available

^a^Recalled twin/triplet

To the best of our knowledge, no patients with severe PID characterized by absent T or B cells at birth have been missed to date among those infants included in the screening program.

### PID Patients

Three patients were found to have repeatedly low levels of both TREC and KREC (*n* = 1) or TREC alone (*n* = 2) (Table 1) and were considered to have a severe immunodeficiency disorder. The first patient had a homozygous splice-site mutation in Artemis (*DLCLRE1C* c.333+2T>G) with absent protein expression and underwent a successful stem cell transplantation at the age of 2 months. The second child shows clinical features of ataxia-telangiectasia and carries compound heterozygous mutations in *ATM*, where the mother contributed a c.3673C>T mutation resulting in a stop codon (p.Gln1225Ter) and the father contributed a c.8653_8654insT mutation resulting in a Val2886CysfsTer10 mutation. The third child was found to have T cell lymphopenia and hypogammaglobulinemia, but genetic analysis (including whole genome sequencing) has not as yet revealed any causative mutation in known PID genes. The results of immunological investigations for the three PID patients identified are given in Supplementary Table 2.

## Discussion

Since 2008, TREC analysis has been the method of choice for screening of newborns for severe forms of primary T cell lymphopenia [6, 7, 16], a method that will capture most, but not all, children with SCID (exceptions being patients with mutations in *ZAP70* [17, 18], *MHC* class II [19–21], and *ADA* (delayed-onset disease [11]). We subsequently developed a triplex method that also includes KREC analysis to assess for potential B cell lymphopenia in DBS samples. This method can successfully identify patients with XLA [12], selected patients with delayed-onset adenosine deaminase deficiency (ADA) [11], Nijmegen-breakage syndrome (NBS) [12], and purine nucleoside phosphorylase (PNP) deficiency [22]. The combined assay has recently also been used to screen patients with SCID [23, 24], ATM [12, 25], Wiskott-Aldrich syndrome (WAS) [26], DiGeorge syndrome [27–29], and trisomy 21 [30], and it has been suggested to be included in routine screening of newborns for primary immunodeficiency [31–34]. KREC levels have also been used to monitor immune reconstitution after bone marrow/stem cell transplantation [35–37].

Ours is the largest study to date using a combined TREC/KREC assay for newborn screening. One SCID patient was identified (and successfully transplanted) in our cohort of 58,000 infants, comparable with the data published by Kwan et al. on a very large US cohort [8]. One patient with ATM was also identified. This disorder is very rare, with an estimated incidence of 1 in 200,000 according to recent figures from the USA [38]. It will be interesting to determine if this finding is due to serendipity or if the disease is more frequent in Scandinavia. Our third patient showed a T cell lymphopenia of unknown cause. Such cases have been previously described in studies in the USA with incidences varying between 3 and 4 per 100,000 children in different states and with a predicted incidence of 1 in 160,000 in a British study (Professor Bobby Gaspar, Great Ormond Street Hospital, personal communication). Recently, during the third year of screening, yet another SCID patient (ADA deficiency) and a patient with severe T cell lymphopenia have been identified.

The number of referrals for flow cytometric analysis in our cohort was low (approximately 1:20,000), markedly lower than that reported in the US study of Kwan et al. [8] (ranging from 1/735 to 1/7500 in the different states). This is a reflection of the strict resampling strategy that was applied to children with low, but not absent TREC or KREC levels. These results also indicate that the addition of KREC testing to the TREC assay in a newborn screening program does not critically drive the demand for second tier testing nor does it increase the fiscal impact of the test itself due to the minimal additional cost of multiplexing.

Prematurity has been considered a predisposing factor for low TREC numbers in studies in American infants [8]. The TREC and KREC levels in our study did show a downward trend with decreasing gestational age, although in the vast majority, the levels were above the cutoff, even in markedly premature infants. This notion was also recently supported [13] in a small Spanish cohort of newborns.

Twins and triplets were markedly overrepresented among the positive samples (*n* = 11/64), potentially reflecting prematurity in the majority of cases (*n* = 8/11). There was a notable difference in TREC and KREC levels within twin pairs returning normal screening results. Although information regarding zygosity was not available, it is expected that one in four pairs is monozygotic and therefore genetically identical, and this observation will thus be further investigated in the future studies.

Thirteen children with low KREC levels were born to 12 mothers who had been treated with azathioprine during pregnancy, three of whom were concomitantly receiving tacrolimus. Two children born to the same azathioprine-treated mother, 2 years apart, both had low KREC levels. Low KREC levels were also recently noted by de Felipe et al. [13] in a small cohort of Spanish children born to mothers treated with azathioprine. As azathioprine may cross the placenta (and is mutagenic), it is generally contraindicated during pregnancy unless used for treatment of severe disease. As B lymphocytes are markedly more sensitive to drug-induced apoptosis than T lymphocytes [39], the selectivity for a reduction in KREC copies alone is not surprising.

We anticipated that some infants with trisomy 21 would screen positive since they may have low B cell and/or T cell production [30]. However, only one of the 43 infants screened during the 2-year period had a KREC level below cutoff, although infants with trisomy 21 had generally lower levels than those in the general newborn population. In Sweden, the estimated frequency of chromosome 22q deletions is around 1/4000, suggesting that some 14–15 children in the screened cohort would suffer from this form of PID. However, less than 10 % of chromosome 22q deletion patients had pronounced T cell lymphopenia (measured as low TREC levels) and an additional 5–10 % had levels close to the cutoff. Thus, we would have expected only one to two children to be identified in our tested cohort, which is not statistically different from the present result (no child identified).

Most of the children in our study were recalled due to low KREC levels. This was expected, as we did not know what the “true” cutoff level for children with XLA would be and thus applied a fairly large safety margin. Ongoing studies in our laboratory, analyzing KREC levels in a large cohort of children with mutation proven XLA, may allow further adjustment of the recall level of KREC, thus reducing the efforts and costs associated with “false” positive results.

Newborn screening for metabolic diseases has undergone major changes during the past decades with inclusion of numerous very rare diseases in the program. We anticipate a similar development in the PID screening field where the KREC may ultimately be added to the TREC assay currently used in several national screening programs. The decision regarding implementation of TREC vs. combined TREC/KREC screening can be guided by considering the advantages and disadvantages of each screening approach. The advantages for adding KREC screening include the identification of children with XLA, late onset ADA, some patients with NBS, and other selected forms of PID whose disease may be undetected by TREC screening alone [8, 23, 40]. In addition, it may assist in distinguishing patients with SCID caused by deficiency in the production of T as well as B cells or only T cells, thus aiding in the diagnostic process. On the negative side, increased costs associated with the additional KREC assay may be considered a disadvantage of the combined screening strategy. With the possibility to multiplex PCR reactions, the individual cost for additional screening markers such as KREC in an existing TREC screening system is negligible (<€0.10 per newborn), but associated costs incurred in follow-up and further testing for infants with abnormal screening results should also be considered. However, it should be noted that the recall rate using the combined TREC/KREC assay is similar to that of TREC screening alone [8], with a lower rate of follow-up FACS studies than in other published studies to date.

As markers for other genetic diseases have been proposed which can be multiplexed with the TREC/KREC system, future efforts may be focused on the extension of existing newborn screening tests for PID [26]. This may potentially include complement deficiencies (using DBS eluates as described by Janzi et al. [41] for C3 deficiencies and Hamsten et al. [42] for C2 deficiencies) and granulocyte defects, thus allowing coverage of a majority of the primary immunodeficiency diseases. Targeted exome sequencing or even whole genome sequencing of newborns may ultimately be considered, which could lead to identification of additional defects of immunity and a large number of other inherited, potentially fatal diseases.

## Electronic Supplementary Material

Below is the link to the electronic supplementary material.

ESM 1 (DOCX 33 kb)

ESM 2 (DOCX 17 kb)
