# Supplementary material for: Newborn Screening for Severe Primary Immunodeficiency Diseases in Sweden—a 2-Year Pilot TREC and KREC Screening Study
Source: J Clin Immunol. 2016 Nov 21;37(1):51–60. doi: 10.1007/s10875-016-0347-5 (PMC5226987; doi:10.1007/s10875-016-0347-5)
Supplement: Supplementary file 1 — (DOCX 33 kb) [file 10875_2016_347_MOESM1_ESM.docx]

**Supplementary Table 1: Characteristics, screening results and further investigation results for 64 newborns recalled for repeat testing.**

| **Cutoff used for each time period** | **Patient** | **Gestational Age** | **Sex** | **Age at 1st sample (days)** | **Mean 1st sample TREC (run in triplicate)** | **Mean 1st sample KREC (run in triplicate)** | **Age at 2nd sample (days)** | **Mean 2nd sample TREC (run in triplicate)** | **Mean 2nd sample KREC (run in triplicate)** | **Diagnosis/Comments** |
| --- | --- | --- | --- | --- | --- | --- | --- | --- | --- | --- |
| cut off TREC ≤15 KREC ≤10  n= 16 582 | 1 | 33 | M | 2 | 77 | 0 | 9 | 137 | 0 | Level day 26 TREC 172 and KREC 240 |
|  | 2 | 39 | M | 2 | 118 | 8 | 10 | 192 | 23 |  |
|  | 3^§^ | 41 | F | 3 | 105 | 3 | 20 | 166 | 52 |  |
|  | 4 | 38 | F (TW I) | 3 | 1 | 18 | n.a. |  |  | Placental abruption, asphyxia, syndromic features, deceased |
|  | 5 | 40 | M | 3 | 226 | 2 | 15 | 352 | 1 | Level day 58 TREC 241 and KREC 18 |
|  | 6 | 40 | M | 3 | 97 | 5 | 12 | 189 | 9 | Azathioprine |
|  | 7 | 39 | F | 3 | 161 | 1 | 15 | 136 | 44 | Azathioprine |
|  | 8 | 25 | F | 3 | 38 | 3 | 13 | 119 | 33 | Premature |
|  | 9 | 37 | M | 2 | 112 | 6 | 18 | 252 | 9 | No additional sampling, no presentation with infections as per available records |
|  | 10 | 39 | M | 4 | 81 | 8 | 19 | 140 | 111 |  |
|  | 11^#^ | 38 | M | 2 | 184 | 9 | n.a. |  |  | No presentation with infections as per available records |
|  | 12 | 34 | M (TW II) | 2 | 13 | 92 | 12 | 162 | 361 | Premature |
|  | 13 | 38 | F | 2 | 12 | 45 | 19 | 57 | 205 |  |
|  | 14 | 39 | M | 3 | 174 | 6 | 17 | 525 | 263 |  |
|  | 15 | 41 | M | 2 | 117 | 6 | 16 | 229 | 98 |  |
|  | 16 | 41 | M | 2 | 79 | 3 | 22 | 191 | 144 | Mercaptopurine |
|  | 17 | 37 | M | 2 | 78 | 2 | 23 | 177 | 156 |  |
|  | 18 | 38 | F | 2 | 77 | 9 | 47 | 127 | 274 |  |
|  | 19 | 40 | F | 2 | 78 | 9 | 32 | 130 | 262 |  |
|  | 20 | 39 | F | 2 | 24 | 8 | 38 | 73 | 377 | Azathioprine |
|  | 21 | 42 | F | 2 | 142 | 5 | 25 | 173 | 110 |  |
|  | 22 | 36 | M | 2 | 43 | 2 | 36 | 84 | 473 | Premature, Azathioprine, Tacrolimus |
|  | 23 | 25 | F (TW I) | 0 | 5 | 23 | n.a. |  |  | Premature, deceased |
|  | 24 | 39 | F | 2 | 141 | 6 | 43 | 251 | 570 | Azathioprine |
|  | 25 | 29 | F (TR II) | 3 | 15 | 29 | 38 | 134 | 352 | Premature, Azathioprine |
|  | 26 | 39 | M | 4 | 234 | 1 | 32 | 410 | 230 |  |
|  | 27 | 41 | M | 2 | 212 | 10 | 24 | 254 | 79 |  |
|  | 28 | 42 | M | 2 | 156 | 9 | 39 | 270 | 197 |  |
|  | 29 | 41 | F | 2 | 248 | 6 | 37 | 379 | 653 |  |
|  | 30 | 42 | M | 2 | 162 | 4 | 28 | 339 | 483 |  |
|  | 31^#^ | 35 | F | 3 | 67 | 3 | n.a. |  |  | Premature, no presentation with infections as per available records |
|  | 32 | 40 | F | 3 | 271 | 6 | 27 | 355 | 484 |  |
|  | 33 | 32 | M | 3 | 13 | 13 | n.a. |  |  | Premature. Level day 772 TREC 86 and KREC 246 |
|  | 34 | 41 | M | 2 | 103 | 8 | 23 | 211 | 323 |  |
|  | 35 | 38 | F | 2 | 153 | 9 | 22 | 409 | 214 | Azathioprine |
| cut off TREC ≤8  KREC ≤4  n= 28 298 | 36 | 29 | M (TW II) | 2 | 5 | 16 | 11 | 51 | 33 | Premature |
|  | 37 | 37 | F | 2 | 244 | 3 | 24 | 354 | 333 |  |
|  | 38 | 40 | M | 2 | 52 | 3 | 28 | 135 | 172 |  |
|  | 39 | 34 | M | 2 | 0 | 0 | 7 | 0 | 0 | Premature, SCID (Artemis), BMT at 2 months of age, alive and well |
|  | 40 | 27 | M (TR III) | 3 | 5 | 28 | 28 | 26 | 106 | Premature |
|  | 41 | 38 | F | 2 | 134 | 4 | 24 | 225 | 380 |  |
|  | 42 | 39 | M | 2 | 5 | 7 | 20 | 5 | 10 | Ataxia-telangiectasia mutation, IgAD, asymptomatic at 1 year of age |
|  | 43 | 40 | F | 2 | 150 | 4 | 21 | 306 | 264 |  |
|  | 44 | 32 | M | 3 | 51 | 4 | 21 | 56 | 27 | Premature, Azathioprine, Tacrolimus |
|  | 45* | 38 | F (TW I) | 3 | 150 | 6 | 26 | 341 | 230 | Azathioprine |
|  | 46* | 38 | F (TW II) | 3 | 132 | 5 | 26 | 267 | 287 | Azathioprine |
| cut off TREC ≤10 KREC ≤6  n= 13 954 | 47 | 36 | M | 2 | 7 | 205 | 27 | 4 | 732 | Premature, T-cell deficiency & B-cell defect of unknown cause |
|  | 48 | 34 | M | 2 | 91 | 4 | 27 | 133 | 106 | Premature, Azathioprine, Tacrolimus |
|  | 49 | 28 | M | 2 | 9 | 12 | n.a. |  |  | Premature, deceased |
|  | 50 | 38 | M | 2 | 47 | 4 | 25 | 231 | 176 |  |
|  | 51 | 42 | F | 2 | 139 | 3 | 28 | 315 | 326 |  |
|  | 52 | 32 | F (TW II) | 2 | 13 | 5 | 10 | 113 | 45 | Premature |
|  | 53 | 24 | F (TW II) | 2 | 9 | 13 | 69 | 230 | 421 | Premature |
|  | 54 | 32 | M | 2 | 88 | 6 | 55 | 224 | 342 | Premature |
|  | 55 | 36 | M | 2 | 192 | 5 | 26 | 260 | 350 | Premature |
|  | 56 | 24 | M | 2 | 9 | 79 | 64 | 12 | 441 | Premature |
|  | 57 | 25 | M | 2 | 10 | 43 | 113 | 115 | 1221 | Premature |
|  | 58^#^ | 25 | M | 2 | 24 | 5 | n.a. |  |  | Premature, no presentation with infections as per available records |
|  | 59^#^ | 28 | F | 8 | 120 | 6 | n.a. |  |  | Premature, Trisomy 21, no presentation with infections as per available records |
|  | 60 | 38 | M | 2 | 79 | 1 | 22 | 163 | 356 |  |
|  | 61 | 41 | M | 2 | 372 | 5 | 33 | 461 | 417 |  |
|  | 62 | 23 | F (TW II) | 2 | 7 | 11 | 17 | 53 | 16 | Premature |
|  | 63 | 38 | M | 2 | 185 | 5 | 22 | 333 | 471 |  |
|  | 64^§^ | 40 | F | 4 | 109 | 6 | 20 | 209 | 50 | Azathioprine |

**siblings, ^§^siblings,* ^#^*declined further testing, n.a. = not available, M = male, F = female, BMT = bone marrow transplant, IgAD = IgA deficiency. TREC and KREC levels are expressed as number of copies/3.2mm blood spot.*
