# Supplementary material for: Newborn Screening for Severe Primary Immunodeficiency Diseases in Sweden—a 2-Year Pilot TREC and KREC Screening Study
Source: J Clin Immunol. 2016 Nov 21;37(1):51–60. doi: 10.1007/s10875-016-0347-5 (PMC5226987; doi:10.1007/s10875-016-0347-5)
Supplement: Supplementary file 2 — (DOCX 17 kb) [file 10875_2016_347_MOESM2_ESM.docx]

**Supplementary Table 2: Immunological investigation results for PID patients.**

|  | **Age-matched**  **reference range** | **Patient 1**  Artemis Deficiency | **Patient 2**  Ataxia-telangiectasia | **Patient 3**  Unknown genetic defect |
| --- | --- | --- | --- | --- |
|  |  |  |  |  |
| **Immunoglobulins** |  |  |  |  |
| IgA (g/L) | - 1. – 0.20   2. – 0.25 | <0.08 | <0.06 | <0.08 |
| IgG (g/L) | 4.0 – 10.0  2.50 – 9.0 | 9.8 | 2.88* | 3.0 |
| IgM (g/L) | 0.15 – 1.0 | <0.10* | 0.34 | 0.10* |
|  |  |  |  |  |
| **Lymphoctye subsets** |  |  |  |  |
| CD3+ T cells |  |  |  |  |
| (x10^9^/L) | 2.3 – 7.0 | <0.01* | 0.54* | 0.73* |
| (%) | 60 – 85 | 1* | 51* | 23* |
| CD3+/CD4+ T cells |  |  |  |  |
| (x10^9^/L) | 1.7 – 5.3 | NA | 0.39* | 0.5* |
| (%) | 41 – 68 |  | 37* | 16* |
| CD3+/CD8+ T cells |  |  |  |  |
| (x10^9^/L) | 0.4 – 1.7 | NA | 0.13* | 0.18* |
| (%) | 9 – 23 |  | 12 | 6* |
| CD4+/CD8+ |  |  |  |  |
| ratio | 1.3 – 6.3 | NA | 3.08 | 2.8 |
| CD19+ B cells |  |  |  |  |
| (x10^9^/L) | 0.6 – 1.9 | <0.01* | 0.09* | 1.73 |
| (%) | 4 – 26 | <0.5* | 8 | 52* |
| CD16+/CD56+ NK cells |  |  |  |  |
| (x10^9^/L) | 0.2 – 1.4 | 0.39 | 0.48 | 0.66 |
| (%) | 3 – 23 | 90* | 41* | 20 |
|  |  |  |  |  |

** denotes result outside of the age-matched reference range, NA = not analysed*
